# Isolated Focal Myositis of the Flexor Pollicis Longus Muscle: A Case Report

**DOI:** 10.7759/cureus.92489

**Published:** 2025-09-16

**Authors:** Vishwas Kadambila, Salauddin Arif K, Sushil KP, Sudharshan Abhishek B S, John Joe Jacob

**Affiliations:** 1 Orthopaedics, Kanachur Institute of Medical Sciences, Mangalore, IND; 2 Orthopaedics, Kanachur Institue of Medical Sciences, Mangalore, IND

**Keywords:** focal myositis, fpl, inflammatory myopathy, intermuscular edema, pseudotumor

## Abstract

Focal myositis is an uncommon inflammatory myopathy that often mimics other pathologies such as soft tissue tumors, infections, or compartment syndromes. Upper limb involvement, especially limited to a single muscle like the flexor pollicis longus (FPL), is rare and diagnostically challenging. We report a case of a 23-year-old woman who presented with progressive atraumatic swelling of the volar forearm. Ultrasound revealed hypertrophy and heterogeneity of the FPL muscle with intermuscular edema. Laboratory markers were within normal limits. A provisional diagnosis of focal myositis was made. The patient gave a favorable response to conservative treatment with non-steroidal anti-inflammatory drugs (NSAIDs) and wrist splinting, along with activity modification. This case underscores the importance of clinical suspicion and radiologic correlation in avoiding misdiagnosis and unnecessary invasive intervention.

## Introduction

Focal myositis is a rare, benign inflammatory condition affecting individual muscles, often presenting as a pseudotumor. It was first described in 1977 by Heffner and remains poorly understood. Focal enlargement within a single muscle is seen with most cases involving lower limb muscles [1]. Upper extremity cases are infrequent, and involvement of the flexor pollicis longus (FPL) muscle is exceedingly rare.

Due to its atypical presentation, focal myositis may be confused with neoplastic, infectious, or compartmental pathologies. Misdiagnosis can lead to unnecessary biopsies or surgical interventions. This report presents a case of isolated FPL myositis, which responded to conservative management, to highlight strategies involved in diagnosis and reinforce the role of high-resolution ultrasonography in guiding management. 

## Case presentation

**Figure 1:**
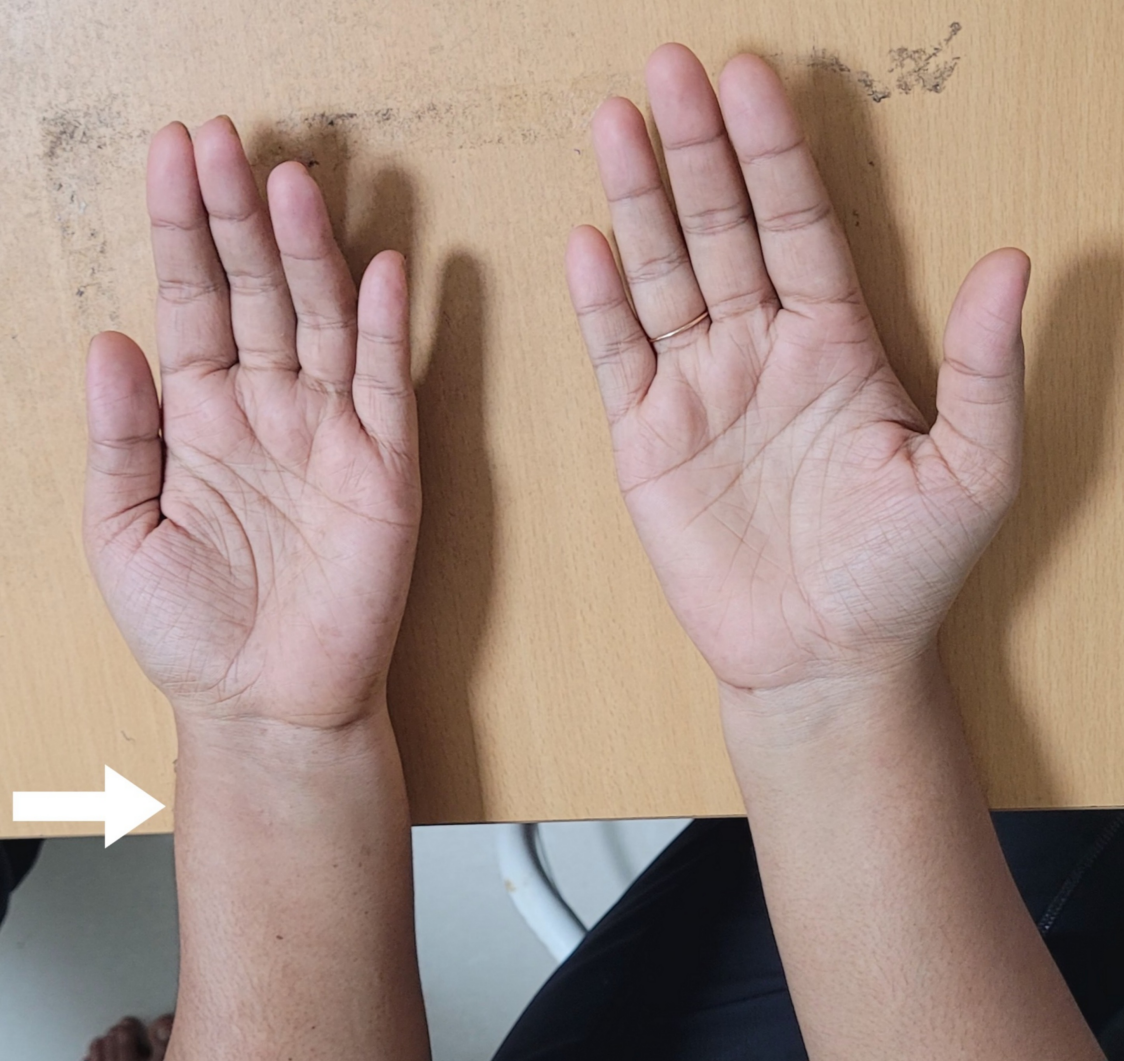
Clinical Photograph Asymmetry visualised between the left and right forearms due to localized swelling over the left volar forearm.

A 23-year-old right-handed female homemaker presented to us with a five-day history of progressive swelling and discomfort in her left forearm. There was no preceding strenuous activity, trauma, fever, systemic illness, or known autoimmune disorder. Clinical examination revealed soft swelling over the distal volar forearm, predominantly anterolaterally (Figure 1).

The area was mildly tender, without warmth, fluctuation, or skin changes. The range of motion of the wrist and fingers was preserved. Passive stretch did not provoke pain. Neurovascular examination was normal, with intact sensation and distal pulses.

Diagnostic assessment

High-resolution ultrasonography of the left forearm was performed by the radiologist on the day of presentation. It revealed a hypertrophic and hypoechoic FPL muscle with internal heterogeneity. There was perimuscular fluid tracking along the intermuscular fascial planes, consistent with edema. A small fluid collection was noted at the myotendinous junction. Flexor tendon sheaths appeared normal. No abscess or mass was detected (Table 1).

**Table 1:** Ultrasonography (USG) Report Indicating Myositis With Forearm Intermuscular Edema

Structure	USG Finding
Flexor Pollicis Longus (FPL)	Heterogeneous and Bulky
FPL Myotendinous junction	Minimal Fluid Noted
Anterolateral Distal Forearm Muscle Compartment	Intermuscular Edema Noted
Tendon Sheaths at Wrist	Normal

Laboratory investigations, including complete blood count, erythrocyte sedimentation rate, C-reactive protein, and creatine kinase, were within normal ranges (Table 2).

**Table 2:** The Patient's Laboratory Parameters The reports were found to be within normal limits.

Test	Result	Normal Reference Range	Unit
Hemoglobin (Hb)	14.5	13.0 - 17.0	g/dL
Total Leukocyte Count (WBC)	6,500	4,000-11,000	/mm^3^
Neutrophils (%)	60	40 - 75	%
Lymphocytes (%)	30	20 — 45	%
Monocytes (%)	5	2 - 10	%
Eosinophils (%)	3	1 - 6	%
Basophils (%)	2	0 - 2	%
Platelet Count	250,000	150,000 - 400,000	/mm^3^
Erythrocyte Sedimentation Rate (ESR)	10	O - 20 (M); O - 30 (F)	mm/hr
C-Reactive Protein (CRP)	1	< 5	mg/L
Creatine Kinase (CK /CPK)	120	30 - 200	U/L

MRI, biopsy, nerve conduction study, and electromyography were considered for further evaluation; however, due to early clinical improvement, along with the patient's unwillingness to undergo additional testing, these were deferred. The diagnosis was supported by the clinical context and characteristic ultrasound findings. 

Differential diagnosis

The following differentials were taken into account and ruled out for the following reasons: a) Early compartment syndrome: Absence of pain on passive finger stretch, normal neurovascular findings, and lack of systemic symptoms argued against this diagnosis; b) Pyomyositis: No fever, leukocytosis, or abscess on imaging; c) Soft tissue tumor: Absence of a discrete mass on sonogram, only muscle hypertrophy seen with no features of invasion of soft tissue planes and preserved surrounding structures; d) Tenosynovitis: Tendon sheaths were preserved, and inflammation was localized to the muscle; e) Eosinophilic fasciitis: No skin tightening, eosinophilia, or systemic manifestations; f) Muscle strain: No history of exertion or trauma and persistent swelling without typical strain patterns.

Therapeutic intervention 

A conservative management plan was initiated. The patient was prescribed oral non-steroidal anti-inflammatory drugs (NSAIDs), placed in a volar wrist splint for immobilization, and advised to limit activity. She was instructed on red flags indicating possible neurovascular compromise. 

Follow-up and outcomes 

At the one-week follow-up, the patient reported substantial improvement in pain and swelling. At three weeks, she remained symptom-free with a complete range of motion and resumed normal activities. She was followed up at three months and again after one year, with no recurrence or residual complaints. She remained functionally independent in all activities of daily living. 

## Discussion

Focal myositis is a localized inflammatory myopathy of unknown etiology with focal enlargement within a single muscle. It has been attributed to immune-mediated, inflammatory, subclinical trauma mechanisms [2]. It can mimic infectious, neoplastic, and compartment pathologies. The rarity of this entity and a nonspecific presentation make diagnosis challenging. Patients usually present with localized muscle swelling, pain, or discomfort. Systemic features such as fever, weight loss, or fatigue are absent [3].

Ultrasound offers a cost-effective, real-time diagnostic modality, particularly in resource-limited settings. It can effectively distinguish focal myositis from abscesses or masses, especially when MRI is not available. In our case, USG findings were characteristic and sufficient for diagnosis in the absence of systemic signs or MRI. While MRI and biopsy are valuable tools for confirming diagnosis and ruling out malignancy or systemic inflammatory myopathies, their use may be guided by clinical course and resource availability [4].

Focal myositis generally responds well to conservative treatment. Non-steroidal anti-inflammatory medication, activity modification, and splinting form the mainstay of treatment. Corticosteroids can be considered in severe or persistent cases, although their use is rarely necessary as spontaneous resolution occurs with conservative measures [5]. In some cases, focal myositis has masked underlying malignancies, emphasizing the importance of clinical vigilance and long-term follow-up [2]. Previous literature supports conservative management in uncomplicated cases, with spontaneous resolution reported [1,3].

In our patient, resolution with NSAIDs and splinting, along with sustained recovery at the end of one year, highlights the benign self-limiting course of the disease. The avoidance of unnecessary invasive procedures underscores the importance of careful clinical assessment and imaging correlation.

## Conclusions

This case illustrates a rare instance of focal myositis involving the FPL muscle. The absence of systemic features and characteristic sonographic appearance allowed for a diagnosis without invasive procedures. Conservative treatment led to complete symptom resolution, and sustained recovery was noted at one year of follow-up. Clinicians should include focal myositis in the differential diagnosis of atraumatic limb swelling, and a structured approach to clinical evaluation, imaging, and management can help avoid unnecessary intervention while ensuring optimal outcomes.
